# Analysis and Correction of Wrong Technical Actions in Juvenile Sports Training Based on Deep Learning

**DOI:** 10.1155/2022/6492410

**Published:** 2022-05-31

**Authors:** Xuefeng Zhao

**Affiliations:** College of Physical Education, Shaanxi Normal University, Xi'an, Shaanxi 710100, China

## Abstract

Scientific analysis of students' incorrect actions in class, as well as timely and effective correction, is frequently an important link in the PE process. At the same time, it is an important symbol for assessing a teacher's teaching level and quality. In this paper, the analysis and correction model of sports wrong technical movements is built using DCNN to address shortcomings in the process of detecting wrong movements in PE and training. This article is based on CNN and has been enhanced by DL. The model learns both manual and DL features; the manual features use an improved dense trajectory, the DL features use CNN based on motion information, and the generalization ability of the kernel support vector machine is used to fuse the two. The simulation results show that the accuracy of the wrong action judgment of this method can reach 92.16 percent, which is 4.6 percent higher than the method of combining NN with region prediction and 5.7 percent higher than the method of detecting image matching score. This method can accurately describe the characteristics of human motion and identify incorrect movements, improve the ability of judging and correcting incorrect movements in sports training, and help athletes improve their sports level.

## 1. Introduction

With the continuous improvement of the technical level of sports, in the teaching process of sports technology, the demonstration of correct movements of sports has become a key research topic in the field of sports teaching [1]. Generally speaking, in the process of mastering motor skills, learners make mistakes, which can be prevented but cannot be absolutely avoided. Thus, correcting wrong actions has become an important task of PE (Physical education) and training [2]. In the actual PE teaching process, due to the obvious differences between students' cognition and understanding levels, some students have more wrong movements and are slow to master the correct movements of PE [3]. At the same time, in all kinds of sports, the technical specifications of movements are more complicated. Especially for some highly technical sports, it is necessary to carry out standardized training with correct movements to improve the level of sports training. In this state, how to effectively correct the wrong movements in sports has become the main problem to be solved urgently in this field. Due to the further development of research in related fields, computer image processing technology has been widely used in sports movement recognition. However, at present, there are various complex movements in sports training, and it is difficult to accurately judge the wrong movements by relying solely on traditional contour detection methods, which leads to the athletes' inability to get movements corrected in time.

With the continuous progress of computer science and technology, artificial intelligence technology [4, 5] has gradually matured. Once the concept of DL (Deep learning) [6, 7] was put forward, a large number of scholars devoted themselves to studying DL and made great progress and innovation. Nowadays, DL has been widely used in the process of human motion recognition and target detection. DL comes from the study of artificial NN (Neural network). In DL, it includes CNN (Convolutional neural network), cyclic NN, Boltzmann machine, etc., but now CNN is the most widely used one. Deep NN can automatically learn data features so as to discover sparse and distributed big data features. However, due to the poor hierarchy of the depth NN constructed in the current research and the failure to further process the action samples, the applicability of this method to the detection of wrong actions in PE teaching and training is poor. This technology can not be directly applied to capture human motion data. Scientific analysis of students' wrong actions in teaching, and timely and effective correction, is often an important link in the process of PE. It is also an important symbol to measure a teacher's teaching level and teaching quality. Based on the idea of improving the teaching quality, this paper improves the shortcomings of the current methods one by one, and builds a technical action analysis and correction model of sports errors based on DCNN (Deep convolution neural network). In order to quickly and accurately detect the wrong movements in PE and training and further improve the sports level of athletes. The innovations of this article are as follows:

1. In view of the shortcomings in the process of detecting the wrong actions in PE and training, this paper is based on CNN and improves it through DL, adding a batch classification layer in the middle of the convolution layer and pool layer, and processing the wrong action samples in PE and training through batch normalization. Given the previous frame, the network can learn to predict the future moving frame and train the time encoder of human motion.
2. In order to meet the needs of large-scale data sets and small-scale motion recognition at the same time, a human motion recognition algorithm based on multifeature fusion and motion information is designed. Through data enhancement, feature optimization, and transfer learning, the detection model in this paper is optimized to improve the detection accuracy and realize the real-time detection of the target. And through self-designed visual calibration objects, the uniqueness detection of multiperson identity can be realized. Experiments show that the algorithm can effectively identify large-scale data sets and small-scale actions.

The article is divided into five parts, which are arranged as follows: The first chapter is the introduction. This part summarizes the background and research significance of this paper and gives the organizational structure and innovation of this paper. The second chapter is related work. This chapter briefly describes the research status of motion recognition methods and target detection and analysis at home and abroad. And the research work and methods of this paper are introduced. The third chapter is divided into two parts.  Section 3.1 outlines the concept of DL and related theoretical basis and introduces the related content of human motion detection based on DL.  Section 3.2 puts forward and constructs the technical action analysis and correction model of sports errors based on DCNN. In chapter 4, the model proposed in this paper is simulated for many times, and the results are analyzed. The fifth chapter is the conclusion. This chapter summarizes the research work of this paper and looks forward to the future.

## 2. Related Work

Accurate recognition and analysis of human body posture can provide effective data support for sports training. By obtaining the relevant data of human movement and correcting the details of athletes' movements with the data of standard database, the athletes' sports level can be improved. Because of the far-reaching development significance, the detection and correction of wrong movements in sports have also become the focus of research in the industry, which has received extensive attention, and many detection methods have also appeared.

Miao et al. designed an action recognition method based on depth images. The algorithm projects the depth image in three projection planes, extracts features from the three projection images, and uses these features to train the extreme learning machine classifier. The algorithm is computationally efficient, but its performance is not ideal for small-amplitude action recognition [8]. Arrieta et al. proposed a temporal deep belief network that can complete online human action recognition [9]. Kluding et al. proposed a gesture feature extraction and recognition method based on acceleration trajectory images. The algorithm converts unknown gesture trajectory features into low-dimensional subfeature sequences by establishing an acceleration gesture trajectory map, which improves the accuracy and time efficiency of gesture recognition [10]. Ullah et al. model human motion information through asymmetric systematic bias [11]. The recognition algorithm proposed by Yao et al. achieves high recognition accuracy for small-amplitude movements, but the amount of features to be analyzed is large, which is difficult to apply to large-scale data sets [12]. Yassine et al. used two-dimensional wavelet packet technology and spatial clustering technology to analyze human behavior and actions and improved the accuracy of wrong action detection by analyzing PE training images. However, the features extracted by the two-dimensional wavelet packet technology are not comprehensive enough, so the subsequent detection results deviate greatly from the actual ones, while the spatial clustering technology is too extensive, ignoring the detailed features of the image edges, and the detection results are prone to errors [13]. Belczak et al. proposed a 3D modeling and detection method for wrong action images in sports without background information [14]. Department et al. proposed an image reconstruction method for wrong action images in sports based on feature moving frame differential scanning and adaptive compensation. In this method, infrared projection and edge contour feature extraction are performed on the wrong action images collected in sports, and the box filtering method is used for image filtering to improve the accuracy of image detection. This method is susceptible to disturbances, resulting in a high error rate for false action detection [15]. Mi et al. proposed a 3D modeling detection method for wrong actions in sports based on 3D contour feature decomposition [16]. The method proposed by Cai et al. uses edge contour reconstruction to repair the missing information of the image and uses the zero-cross point feature segmentation and corner detection method to perform adaptive positioning and detection of wrong actions in sports, image classification, and screening. This method improves the detection and recognition ability of wrong actions [17].

In this paper, the traditional methods of moving target detection and motion recognition are summarized, and their advantages and disadvantages are analyzed. Given the shortcomings in the current detection process of incorrect movements in PE teaching and training, a model for analyzing and correcting incorrect technical movements in PE is built using DL. The model learns both manual and DL features; the manual features use an improved dense trajectory, the DL features use CNN based on motion information, and the generalization ability of the kernel support vector machine is used to fuse the two. The detection model in this paper is optimized through data enhancement, feature optimization, and transfer learning to improve detection accuracy and achieve real-time detection of the target. Furthermore, the detection of multiperson identity uniqueness can be realized using self-designed visual calibration objects. This method can accurately describe the characteristics of human motion, identify incorrect movements in sports training, and improve the ability to judge and correct incorrect movements. It has theoretical and practical implications in the field of sports action recognition and correction.

## 3. Methodology

### 3.1. Human Motion Detection Based on DL

Artificial NN is an algorithm model inspired by nature and constructed by simulating human brain nerve activity. The basic unit of artificial NN is the neuron, which is also the most basic unit for processing all information. By simulating the process of the human neuron processing external stimuli, the neuron appears as a many-to-one nonlinear mapping unit. DL comes from the research of artificial NN [18]. There are four basic models of DL. Specifically: (1) Automatic encoder, sparse automatic encoder, and noise reduction automatic encoder with their variants. (2) Limiting Boltzmann machine. (3) Convolution Boltzmann machine. (4) Circulation NN. Action recognition is influenced by different lighting conditions, diverse perspectives, complex backgrounds, and great changes within a class. In this field, the improved dense trajectory method is the best method of traditional methods. It has good stability and the highest reliability, but the algorithm is very slow. The appearance of DL provides a new idea for action recognition and achieves good results.

**Figure 1:**
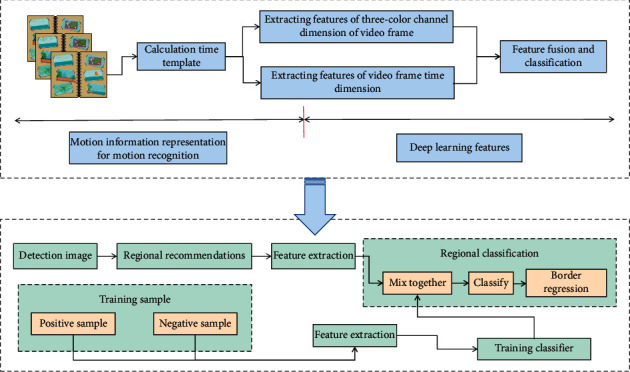
Target detection process based on deep learning.

DCNN is a multilayer backpropagation artificial NN designed for shape recognition in DL. Each layer of neurons is linked to the local visual field area where the front layer network intersects, but there are no connections between neurons in the same layer. DCNN is primarily a type of NN that employs DL to improve CNN by displaying changeable levels and backward propagation direction, allowing for error action detection [19]. Weight sharing and local connection are features of DCNN, which greatly reduce network complexity and improve algorithm performance. As a result, DCNN is the most popular NN [20]. The pooling layer is usually connected after the convolution layer. Then the convolution layer is alternated with the full connection layer. Convolution kernels of various sizes can be used to improve the overall generalization performance of the network during the convolution process. A classifier will be connected at the end of the network to classify the input data. The process of object detection based on deep learning is shown in Figure 1.

DCNN has certain advantages. It includes the following: (1) Minimize pretreatment. Most of the input data are raw data that have not been processed many times, and there is no accurate mathematical expression between input and output. Its feature extraction is trained within the network, which avoids the subjectivity and limitations of manual feature extraction. (2) DCNN is influenced by time delay NN and adopts the network structure of weight sharing, which reduces the computational complexity in the process of network training and makes it more suitable for time series data. DCNN weights are shared and the calculation during training is relatively simple, which can be widely used in the process of time series data analysis, and it can change the capacity of the wrong action detection model in PE and training through the depth and breadth of the network structure. The structure of the dual-flow network is that two NN mix features before the final decision. For the spatial information feature extraction network, only the intercepted video frames need to be grouped into network training to get the spatial features. For the time information feature, the optical flow information of several consecutive frames is usually selected as the input of the network and then added to the network for training. The intrinsic characteristics of the signal, the statistical characteristics of one part may be the same as those of other parts. Therefore, a small subimage can be randomly selected from the image for feature learning, and then the feature can be used as a filter to scan the whole image, and a different feature activation value can be extracted at each position of the whole image, thus realizing feature extraction of the image.

Intercepting video frames according to a set frame rate is the simplest way to deal with video for motion recognition. Processing video frames requires significantly less computation than directly processing video data. Pooling the feature map obtained from the convolution layer through the pooling layer yields the corresponding pooled feature map. A feature extractor is analogous to a convolution kernel. Different convolution kernels can be designed to extract features at different levels in the image, and all of the features processed by convolution kernels are then combined as the output of the convolution layer, so convolution kernel design is crucial. Convolution kernel size, number, and step size can all be customized. The convolution operation is carried out in the cube by combining several consecutive video frames into a video frame cube. The sequential convolution operation involves performing three consecutive video frames at the same time, and the time dimension of the convolution kernel is three, making it a three-dimensional convolution kernel. The video frame cube's convolution kernel weights are shared. A variety of convolution kernels are used for convolution layer operation in order to better obtain the information in the video frame.

### 3.2. Analysis and Correction Model of Technical Action of Sports Errors

DCNN weight sharing has a network structure that is more similar to biological perception NN, and it can adjust model capacity by changing the depth and breadth of the network structure, which has strong statistical stationarity and pixel local correlation for natural images. Convolution and pooling are two important DCNN links. As a result, one of the key factors that determine the performance of a DCNN structure is the choice of convolution kernel size and sliding step size, pool kernel size, and sliding step size. The model's parameter training is also a critical factor in determining the network structure's performance. Because CNN requires a large number of training parameters, the model's complexity is high, which could lead to the trained model's error rate on the training set decreasing while the error rate on the verification set increases. The main reason is that in the training process, every input must be considered, and there are numerous parameters to consider. A small change in the input data has a big impact on the outcome, so keeping the parameters as small as possible is the problem that needs to be solved right now.

**Figure 2:**
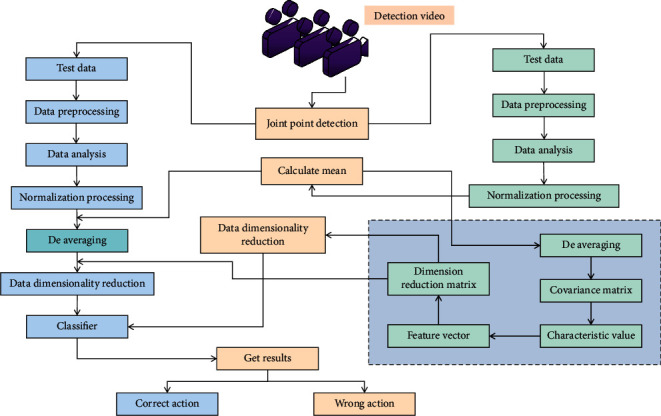
Sports action detection and analysis process.

In this paper, the regularization method is frequently used in the full connection layer to prevent overfitting due to the small size of the data set used in the DCNN process. Because of the randomness of this method, the network structure corresponding to each transmitted data set will be inconsistent, but all network weights will be shared, greatly improving the stability of the wrong action detection model in PE and training and making it easier for neurons to adapt to one another. Every pixel in a video frame or image is made up of three primary colors. The amount of data read at one time during network training is large, which has a significant impact on the computer's computing speed. When training the model, end-to-end training can be done when the two pieces of information match, and the corresponding real frame can be chosen from a list of default frames with various aspect ratios, scales, and positions. The batch normalization algorithm is used in the batch normalization layer in this paper, which integrates the network layer input processing operation into the wrong action detection of PE and training. It processes the wrong action samples of PE and training through microbatch normalization. The flow of sports movement detection and analysis using this method is shown in Figure 2.

The size of convolution kernel has obvious influence on image processing. If the convolution kernel used is too small, the enhancement effect of the action information of interest to the image is not obvious, but it may increase the noise in the same frequency band and cover up some original details of the image. If the convolution kernel used is too large, the calculation amount of convolution will also increase, resulting in higher computational complexity. According to a large number of applications of DCNN structures in human motion recognition, this paper finds that the convolution kernel size is 3 × 3 convolution network structure. ResNet residual network is an improvement to solve the problem of deep network structure degradation. ResNet is based on the cross-layer connection structure of Highway Network. The number of layers of residual network is usually very large, up to 100 layers, but this fast connection method makes the actual depth of the network not too deep. Experiments show that residual network can well solve the problem of deep network degradation. When the auxiliary features are used as input to pass through the last hidden layer of the proposed model and connected with the auxiliary output points, and the extracted features are closer to the output of the auxiliary features in the training stage of the proposed network model, the regularization of the model is completed. The convolution layer of DCNN applies the weight sharing method, and at the same time, it reduces the structural parameters and difficulty, prevents NN from overfitting in the early stage, makes it have better generalization ability, and ensures the stability of NN through pooling. Suppose the formula of the fully connected layer feature output *x*^(*l*)^ of the *l* layer is as follows:

(1) $$\begin{array}{c} x^{\left(l\right)}=f\left(w^{\left(l\right)}x^{\left(l-1\right)}+b^{\left(l\right)}\right). \end{array}$$

Among them, *w*^(*l*)^ represents the weight parameter, and *b*^(*l*)^ is the bias term. The Softmax regression classifier needs to iteratively update and learn, and the functions to be learned are as follows:

(2) $$\begin{array}{c} h_{w}\left(\overset{⟶}{x}\right)=\frac{1}{\sum_{i=1}^{k}e^{\overset{⟶}{w_{i}}\cdot \overset{⟶}{x}+b_{i}}}\left[\begin{array}{c} \overset{⟶}{w_{1}}\cdot \overset{⟶}{x}+b_{1}\\ \overset{⟶}{w_{2}}\cdot \overset{⟶}{x}+b_{2}\\ \ldots \\ \overset{⟶}{w_{k}}\cdot \overset{⟶}{x}+b_{k} \end{array}\right]. \end{array}$$

Among them, *k* represents the number of categories to be classified, and *b*_*i*_ and $\overset{⟶}{w_{i}}$ represent the offset vector and weight vector corresponding to the i th category. (3) represents the probability value that the sample $\overset{⟶}{x}$ is the *j* class.

(3) $$\begin{array}{c} P\left(y=j|\overset{⟶}{x}\right)=\frac{\overset{⟶}{w_{j}}\cdot \overset{⟶}{x}+b_{j}}{\sum_{i=1}^{k}e^{\overset{⟶}{w_{i}}\cdot \overset{⟶}{x}+b_{i}}}\sum_{j=1}^{k}P\left(y=j|\overset{⟶}{x}\right)=1. \end{array}$$

After training and learning to get $\overset{⟶}{w_{i}}$ and *b*_*i*_, the objective loss function can be expressed as follows:

(4) $$\begin{array}{c} J\left(w,b\right)=-\frac{1}{m}\sum_{j=1}^{m}\sum_{l=1}^{k}1\left\{y^{\left(j\right)}=l\right\}\text{log}\frac{e^{\overset{⟶}{w_{l}}\cdot \overset{⟶}{x}+b_{l}}}{\sum_{i=1}^{k}e^{\overset{⟶}{w_{i}}\cdot \overset{⟶}{x}+b_{i}}}. \end{array}$$

Among them, m represents the number of samples in the training set, k represents the number of classification categories, and 1{·} is an indicative function. When *y*^(*j*)^=*l*, the function value is 1. Otherwise, it is 0. Optimized by an autoencoder for high-dimensional input data *x* ∈ *R*^*N*^:

(5) $$\begin{array}{c} \underset{f,g}{\min}\left\|x-f\left(g\left(x\right)\right)\right\|. \end{array}$$

Among them, the encoder *y*=*g*(*x*) maps the input data to the low-dimensional space *y* ∈ *R*^*M*^, *N* > *M*. Let *x* ∈ *R*^*N*^ be the observations of time *t*. The optimization function of the time encoder is as follows:

(6) $$\begin{array}{c} \underset{f,g}{\min}\left\|X_{\left(t+1\right):\left(t+\Delta t\right)}-f\left(g\left(X_{\left(t-\Delta t+1\right):t}\right)\right)\right\|, \end{array}$$

where the encoder *y*=*g*(*X*_(*t* − Δ*t*+1):*t*_) maps the input data to the low-dimensional space *y* ∈ *R*^*M*^, (*N* × Δ*t*)*n*!/*r*!(*n* − *r*)! > *M*. The decoder ${\hat{X}}_{\left(t+1\right):\left(t+\Delta t\right)}=f\left(y\right)\in R^{N\times \Delta t}$ is used to map back to the data space.

Neurons appear with a certain probability in each layer of the network structure, causing the training network to change every time, reducing the correlation between neurons and increasing the generalization and robustness of the entire network. The depth of NN affects the detection accuracy of incorrect movements in sports, and the features are related to representation ability. The NN depth will calculate all of the features of incorrect movements, and the deeper the final output, the better the feature extraction ability. The extreme learning machine is divided into two layers: the first calculates two feature cores, then fuses the two to form a fused feature core, and finally outputs the predicted scores of the three feature cores. All of the predicted scores are mapped to the final action classification by the second training classifier. This method's manual and DL features complement each other, and the video's human motion information is described from various perspectives. The independent batch normalization process replaces the joint normalization process of each dimension of data, and the formula is as follows:

(7) $$\begin{array}{c} {\overset{⌢}{X}}^{\left(k\right)}=\frac{x_{i}^{\left(k\right)}-E\left[x^{\left(k\right)}\right]}{\sqrt{\mathrm{var}\left[x^{\left(k\right)}\right]}}. \end{array}$$

Among them, the *k* th dimension of the input sample is denoted by *x*^(*k*)^, the expectation is denoted by *E*[*x*^(*k*)^], and the variance is denoted by var[*x*^(*k*)^]. Adding parameters *λ*^(*k*)^ and *β*^(*k*)^ to the *k* dimension of each input sample, the following formula is obtained:

(8) $$\begin{array}{c} y^{\left(k\right)}=\lambda^{\left(k\right)}{\overset{⌢}{X}}^{\left(k\right)}+\beta^{\left(k\right)}. \end{array}$$

Among them, *λ*^(*k*)^ and var[*x*^(*k*)^] are equal and both are variances. The local binary fitting method is used to complete the information collection and the reconstruction of the feature database, and the regional pixel information can be obtained:

(9) $$\begin{array}{c} L=J\left(w,e\right)-\sum_{i=1}^{N}a_{i}\left\{w^{T}\emptyset \left(x_{i}\right)+b+e_{i}+y_{i}\right\}. \end{array}$$

Among them, *J*(*w*, *e*) refers to the repeated pixels of the motion position; *x*_*i*_ and *y*_*i*_ are the correct action and wrong action feature vectors of the *i* th Gaussian unit, respectively; *a*_*i*_ is the standard action configuration sequence; ∅(*x*_*i*_) is the contour feature distribution function. By estimating the probability of the motion feature obtained by dimensionality reduction in the Gaussian unit *i*, we can get the following:

(10) $$\begin{array}{c} r\left(i\right)=\frac{w_{i}p_{i}-v_{i}}{\sum_{i=1}^{k}w_{i}p_{i}\left(v_{i}\right)}. \end{array}$$

Among them, *p*_*i*_ refers to the probability of being assigned to the *i* th Gaussian unit, and *w*_*i*_ is the mixing weight.

A low-dimensional mapping, that is, a fixed-length feature vector, is generated for each *n* × *n* sliding window position, and then these feature vectors are sent to two fully connected layers, respectively, one for border regression and the other for predicting the category of the sliding window target; that is, the transformation parameters of the border position are given. Because the gray-scale data and the original data are identical in video length, data set size, action types, etc., except for the single pixel data of the image, the gray-scale image is chosen to preliminarily determine the training parameters of the network structure. In the process of deepening the network depth, it is easy for the gradient to disappear, which leads to a decline in network performance. To solve this problem, the basic network used to extract features by DCNN is ResNet101, which can extract the subtle features of PE training sample data faster and better. At the same time, in the middle of the convolution layer and pooling layer, the normalized layer and residual block are added in batches through ResNet, which can speed up the network training and adjust the data transmission strategy to further optimize the network performance.

## 4. Result Analysis and Discussion

In DL, the more effective samples are produced, the better the robustness of the trained model will be. Therefore, this paper uses some data enhancement strategies at the beginning of the network and carries out some random operations for each input sample picture, including using the original input sample picture; Sampling the original picture at random; Cut the original picture randomly, etc. After the above operation, the sample sizes of all samples are normalized, and the samples are randomly flipped horizontally with a certain probability. In order to determine the batch size of network training, the network training is carried out at every 10 groups of samples based on the batch data from 10 to 100 under the condition of 10 iterations, 20 iterations, and 100 iterations, respectively. Among them, the parameter settings of DCNN are shown in Table 1.

**Figure 3:**
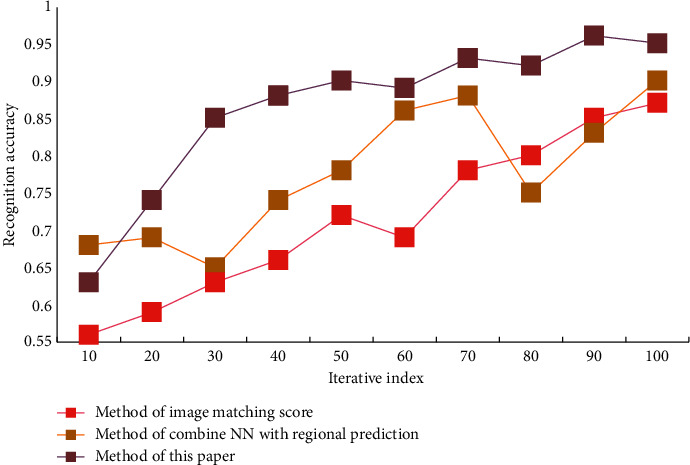
Action recognition accuracy of different algorithms—dataset A.

**Figure 4:**
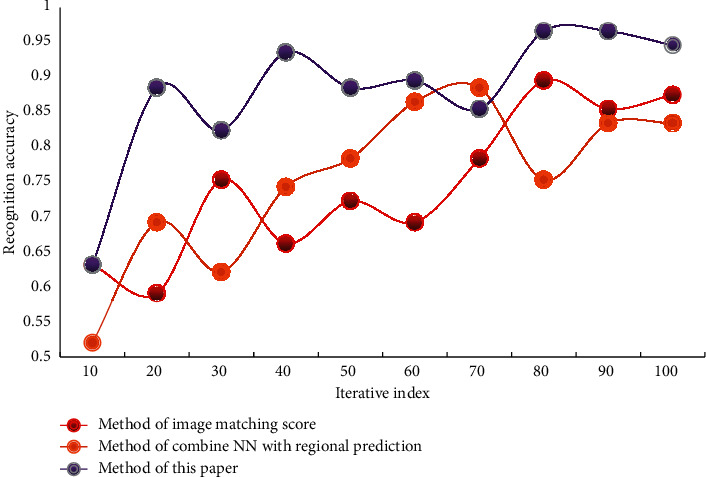
Action recognition accuracy of different algorithms—dataset B.

In order to comprehensively evaluate the performance of this algorithm, experiments were carried out on video dataset A and video dataset B, respectively. Data set A has a low resolution and a large amount of data, which can test the recognition performance of this algorithm for large-scale data sets. The video data set B has a high resolution, including 35 actions, all of which are local actions of the human body, with a small range of actions. This data set can test the recognition effect of this algorithm for small-amplitude actions. During the experiment, the data set used in this paper is a nonpublic data set. All experimental data are divided into two groups on average, one for DCNN training and the other for experimental testing. The accuracy of action recognition of different methods on two data sets is shown in Figures 3 and 4.

**Figure 5:**
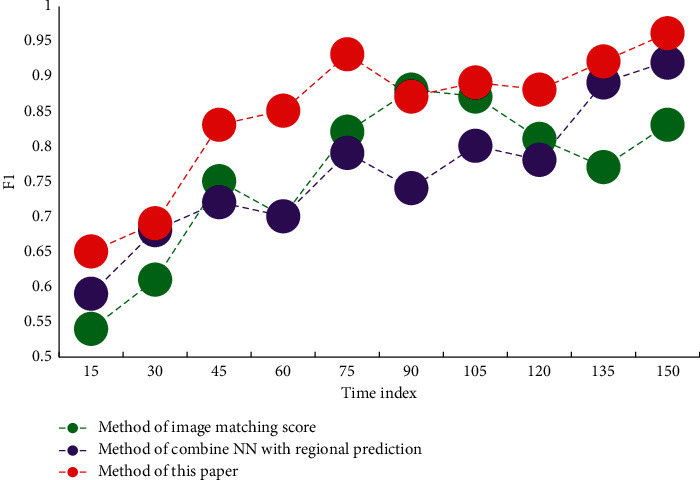
F1 value comparison.

In this paper, we sort the confidence of the default boxes of negative samples, then select some default boxes in the front as samples, and discard all other negative samples, so that the final ratio of positive and negative samples is about 1 : 3. Experiments show that this operation can accelerate the convergence speed, and the training process is more stable, which is a very effective optimization strategy. In this paper, representative F1 indicators are plotted as data as shown in Figure 5.

**Figure 6:**
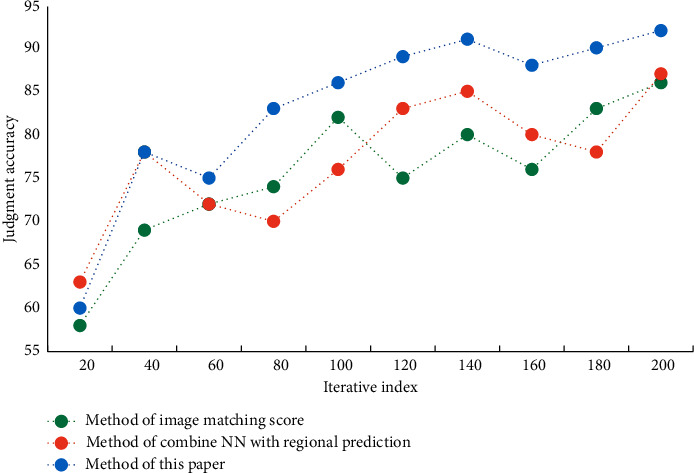
The effect of different algorithms for detecting wrong actions in PE training—dataset A.

**Figure 7:**
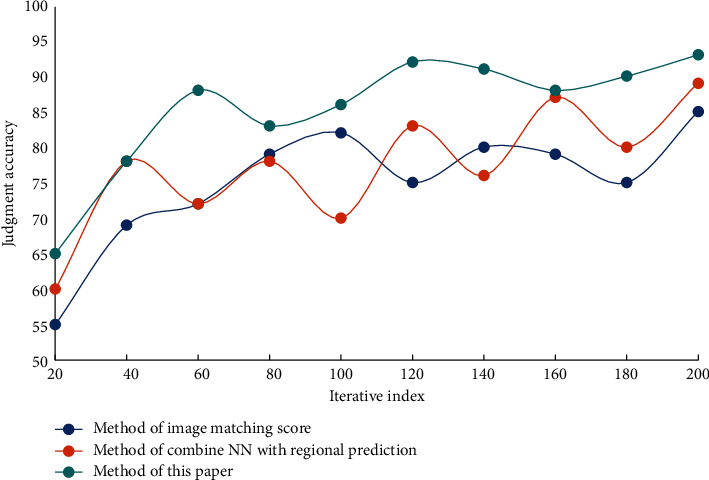
The effect of different algorithms for detecting wrong actions in PE training—dataset B.

In the process of recognition, the processing of different frames will also occupy different sizes of computing resources. In order to reduce the use of computing resources, before the network training, firstly, the key area of human motion is detected by the method of target detection, and this area is cut out, which is used as new data to join the network for training. The method in this paper, the method of combining NN with regional prediction and the method of image matching score are used to detect the wrong actions in the process of PE teaching and training, and the effects of the three methods in detecting the wrong actions in PE teaching and training are compared with the increasing number of experiments. The effects of three different methods on detecting the wrong actions in PE teaching and training on two data sets are shown in Figures 6 and 7.

From the data analysis in Figure 7, it can be seen that the method in this paper has a certain accuracy in judging wrong actions. Among them, the error judgment accuracy of this method is about 92%, the error judgment accuracy of the method combining NN with regional prediction is about 87%, and the error judgment accuracy of the image matching score method is about 86%. The problem of small target detection is addressed in this paper by adding another layer to the feature selection layer, selecting lower-level features, and retaining more lower-level detail features. The video data is preprocessed as network input and the network parameters are effectively trained. The extracted motion features are then convolutioned and pooled to add more time and space dimension information. Finally, it passed through the softmax classifier, which improved the accuracy of human motion recognition and better adapted to different changes in video data scenes, thanks to two layers of full connection. Six test indexes, namely accuracy, recall, sensitivity, complexity, specificity, and positive prediction rate were used for quantitative comparison in order to verify the effectiveness and robustness of this method in the detection of incorrect movements in sports training. The comparison results are shown in Table 2.

The data analysis in the table shows that among the three methods of detecting incorrect actions in PE teaching and training, the six parameters of this method are better than the method of combining NN with regional prediction and the method of image matching score. The model in this paper has fewer parameters, which reduces overfitting and reduces structural redundancy, resulting in a faster convergence speed and a shorter training period. It improves the accuracy of recognizing sports incorrect actions and reduces running time to some extent. This confirms the superior performance of the DCNN proposed in this paper.

## 5. Conclusions

It is natural for students to make mistakes when learning new movements in technology classes, and teachers should prevent and correct these errors. Preventing and correcting incorrect movements in technology teaching is a necessary condition for effectively exercising and avoiding sports injuries. However, due to the variety of complex movements in sports training, it is difficult to accurately judge the incorrect movements using traditional contour detection methods, resulting in athletes being unable to correct the movements in a timely manner. Traditional methods, on the other hand, are unable to accurately capture the incorrect movement characteristics of PE teaching and training, resulting in decreased detection accuracy. This paper proposes a DCNN-based detection model of incorrect actions in PE teaching and training based on this information. The model learns manual and DL features, with the manual features using an improved dense trajectory and the DL features using a CNN based on motion information, and the two features are fused using the generalization ability of a kernel support vector machine. Simulation experiments are conducted in this paper to demonstrate the efficacy of the method proposed in this paper for detecting incorrect actions in PE teaching and training. The results show that the accuracy of wrong action judgment can reach 92.16 percent, which is higher than the 4.6 percent accuracy of the method that combines NN and region prediction and the 5.7 percent accuracy of the method that detects image matching score. The accuracy and robustness of human motion feature analysis are improved because the network structure used in this paper can directly classify actions on features without fine-tuning. The findings show that this method can accurately detect athletes' incorrect movements during PE and training, control detection errors, and judge incorrect movements quickly and accurately. For the field of sports action recognition and correction, this study has some theoretical and practical relevance. Despite the fact that this paper yielded some research findings, it still has some flaws that need to be investigated and improved. We will continue to research how to improve timeliness and immediacy while also increasing recognition accuracy in future work. In PE teaching and training, provide strong technical support for detecting incorrect movements.

## Figures and Tables

**Table 1:** Parameter settings of DCNN.

Number of plies	Function
1	Input layer
2	Convolution layers 3-64
3	Maximum pool layer
4	Convolution layers 3-128
5	Convolution layers 3-128
6	Maximum pool layer
7	Convolution layers 3-256
8	Convolution layers 3-256
9	Maximum pool layer
10	Convolution layers 3-512
11	Convolution layers 3-512
12	Maximum pool layer
13	Convolution layers 3-512
14	Convolution layers 3-512
15	The full connection layer 3072
16	Output layer

**Table 2:** Detection results of wrong actions in PE training with different methods.

Test index	Methods of this paper	Method of combining NN with regional prediction	Method of image matching score
Accuracy rate	0.927	0.879	0.861
Recall rate	0.948	0.906	0.874
Sensitivity	0.948	0.891	0.876
Complexity	0.642	0.726	0.774
Specificity	0.026	0.054	0.113
Positive predictive rate	0.098	0.214	0.239

## Data Availability

The data used to support the findings of this study are available from the author upon request.
